# The epidemiology of carbapenem-non-susceptible *Acinetobacter* species in Europe: analysis of EARS-Net data from 2013 to 2017

**DOI:** 10.1186/s13756-020-00750-5

**Published:** 2020-06-19

**Authors:** Olaniyi Ayobami, Niklas Willrich, Beneditta Suwono, Tim Eckmanns, Robby Markwart

**Affiliations:** 1 Department 3: Infectious Disease Epidemiology, Unit 37: Healthcare-associated Infections, Surveillance of Antibiotic Resistance and Consumption, Robert Koch Institute, Nordufer 20, 13353 Berlin, Germany; 2 Federal Institute for Risk Assessment, Unit Epidemiology, Zoonoses and Antimicrobial Resistance Department Biology Safety, Berlin, Germany

**Keywords:** *Acinetobacter*, *Acinetobacter baumannii*, Carbapenem resistance, Antimicrobial resistance, Surveillance, EARS-Net

## Abstract

**Background:**

Due to limited therapeutic options and their association with high mortality and morbidity, carbapenem-non-susceptible *Acinetobacter* spp. (CNA) are of significant public health importance. This study aimed to describe current epidemiological trends of CNA proportions in Europe and to identify factors that are associated with carbapenem non-susceptibility of isolates from patients with invasive *Acinetobacter* spp. infections.

**Methods:**

Data from routine carbapenem susceptibility testing of 18,412 invasive clinical *Acinetobacter* spp. isolates from 30 European countries in 2013–2017 were analysed using descriptive statistical analyses and uni- and multivariable regression analyses. These data were obtained from the European Antimicrobial Resistance Surveillance Network (EARS-Net).

**Results:**

The population-weighted mean proportion of carbapenem-non-susceptible *Acinetobacter* spp. in Europe is 35.6% (95% confidence interval [CI] 29.7–42.0%). With CNA proportions of 75.5% (95% CI 71.2–79.4%) and 71.5% (95% CI 66.7–75.9%) the burden of CNA is particularly high in Southern and Eastern European regions. In contrast, Northern and Western European regions recorded CNA proportions of 2.8% (95% CI 1.2–6.0%) and 6.3% (95% CI 4.5–8.9%), respectively. Population-weighted mean CNA proportions are especially high in *Acinetobacter* spp. isolates from intensive care units (54.0% [95% CI 47.6–60.3%]). Male gender, age above 20 years and ICU admission were identified as independent factors associated with an increased likelihood of CNA.

**Conclusion:**

The burden of carbapenem-non-susceptible *Acinetobacter* spp. is particularly high in Southern and Eastern Europe. There is a risk that resistance could spread to other parts of Europe. Therefore, increased efforts in infection control and antibiotic stewardship, particularly in Intensive Care Units, are necessary to combat the spread of CNA in Europe.

## Background

*Acinetobacter* species spp. are non-fermenting, largely opportunistic, gram negative bacteria that are ubiquitous in the environment. *Acinetobacter baumannii* complex (*Acinetobacter nosocomialis, Acinetobacter pitti* and *Acinetobacter baumannii*) are the most clinically significant among the over 50 species in the Acinetobacter genus [1]. Of all *Acinetobacter* spp.*, Acinetobacter baumannii* sensu stricto (referred to *Acinetobacter baumannii* in this article) is responsible for about 90% of the *Acinetobacter* spp. clinical infections in humans [1]. Its ability to survive harsh conditions, including desiccation and disinfection, fosters its persistence and spread in hospital environments [2, 3]. The *Acinetobacter* spp. have also evolved multiple resistance strategies, such as over-expression of efflux pumps, reduced permeability and a diverse array of hydrolytic carbapenemases [4], resulting in resistance to carbapenems and other reserve drugs. In addition, *Acinetobacter baumannii* is naturally resistant to several antibiotics, including priority “Watch group” antibiotics as defined by the WHO, such as cephalosporin. It has acquired many more resistance strategies, leading to poorer clinical outcomes across many healthcare settings [5, 6]. Carbapenem-non-susceptible *Acinetobacter* spp. (CNA) is increasingly recognised as a nosocomial pathogen of significant public health importance worldwide because it presents limited therapeutic options, high treatment costs and is associated with high mortality and morbidity [7–9]. In the World Health Organization’s (WHO) recent drug research and development prioritisation exercise for pathogens, CNA emerged as the highest weighted antimicrobial-resistant pathogen [10]. Worldwide, CNA have been implicated in several outbreaks of pneumonia, bloodstream, wound and urinary tract infections, especially among patients with severe morbidities like those on Intensive Care Units (ICU) [11, 12].

According to the 2017 *European Antimicrobial Resistance Surveillance Network (*EARS-Net*)* report, proportions of CNA collected from invasive infections are particularly high in many southern and eastern European countries where resistance proportions often exceed 50% (such as 95% in Greece, 79% in Italy, and 53% in Hungary) [13]. In contrast, western and northern European countries have recorded low proportions of resistance to carbapenems, often less than 5% (such as 3% in the United Kingdom, 4% in Germany and < 1% Norway, Denmark and Sweden).

Despite these European data, a comprehensive epidemiological picture of invasive carbapenem-non-susceptible *Acinetobacter* spp. in European hospitals is still lacking. While published EARS-Net reports focus on analyses of national trends, until now it has not been systematically assessed how the likelihood of carbapenem-non-susceptibility is impacted by patients’ characteristics (such as gender and age) and other factors (such as hospital unit type, season of the year and antibiotic consumption). This study therefore aims to investigate epidemiological trends of CNA proportions in Europe and seeks to identify factors that are associated with carbapenem-non-susceptibility of isolates from patients with invasive *Acinetobacter* spp. infections using EARS-Net data from 2013 to 2017.

## Methods

### Study design and the European antimicrobial resistance surveillance database

We conducted a retrospective observational study on *Acinetobacter* spp. using data retrieved from the EARS-Net database. EARS-Net is a network of European surveillance systems collecting routine clinical antimicrobial susceptibility (AST) data on invasive isolates (blood and cerebrospinal fluid [CSF]) from the 28 European Union countries as well as Norway and Iceland [13]. Detailed information about the EARS-Net methodology is provided in their surveillance reports and protocols [14]. In EARS-Net, no information on the specific species of the *Acinetobacter* spp. isolates are reported. *Acinetobacter* spp. are classified by EARS-Net as sensitive (S), intermediate (I), or resistant (R) to antimicrobial drugs, based on the standards used in the participating laboratories, such as the guidelines of the European Committee on Antimicrobial Susceptibility Testing (EUCAST), Clinical and Laboratory Standards Institute (CLSI) or other national guidelines. In our data set, more than 98% of all isolates with information on the used guideline have their AST interpreted with EUCAST or CLSI. As reported by the ECDC, there is a general consensus between the EUCAST and CLSI interpretation for carbapenem susceptibility testing for Acinetobacter spp. [15].

In addition to S-I-R data, individual laboratories provide further epidemiological information. This includes the date of specimen collection, specimen type (i. e. blood and CSF), care type (such as inpatient or outpatient care), patient gender and patient age as well as the hospital unit (such as ICU or internal medicine unit), where the sample was collected.

### Selection of *Acinetobacter* spp. isolates

We extracted data for *Acinetobacter* spp. from the TESSy database in February 2019 with the approval of the European Centre for Disease Prevention and Control. Only the first isolate from a given patient in the respective year is included in the TESSy database of EARS-Net. We excluded isolates with multiple AST tests against the same antibiotic as well as isolates with the same unique identification number. In the next steps, we excluded isolates from outpatient care and isolates that were neither tested against meropenem nor imipenem. Since the initial pilot study of EARS-Net was conducted in 2012 and included data from only 18 countries, we exclusively analysed data on *Acinetobacter* spp. from 2013 to 2017, when all 30 countries provided data to EARS-Net.

### Outcomes and variables

The primary outcome of interest is the population-weighted proportion of carbapenem-non-susceptible *Acinetobacter* spp. isolates among all *Acinetobacter* spp. isolates, expressed as a percentage (%) and with 95% confidence intervals (95% CI). An *Acinetobacter* spp. isolate was defined as carbapenem-non-susceptible if it was tested resistant or intermediate against meropenem and/or imipenem. Clinical specimens were grouped by sampling site into either blood or cerebrospinal fluid. Patient ages were categorized into six age categories (< 1, 1–19, 20–39, 40–59, 60–79 and ≥ 80 years). Patient genders were classified as female or male. The isolates’ country of origin were grouped into four major European regions (**Northern:** Denmark, Finland, Iceland, Ireland, Norway, Sweden, United Kingdom; **Western:** Austria, Belgium, France, Germany, Luxembourg, Netherlands; **Southern:** Croatia, Cyprus, Greece, Hungary, Italy, Malta, Portugal, Slovenia, Spain; **Eastern:** Bulgaria, Czech Republic, Estonia, Latvia, Lithuania, Poland, Romania, Slovakia). Hospital units were categorised into “ICU”, “Internal medicine”, “Surgery”, “Oncology” and “Others”. To determine potential seasonal trends in the occurrence of CNA, the months in which the sample were collected were categorised into “Warm months” (May – September) and “Cold months” (October – April). In order to investigate the population-weighted proportion of co-resistance to gentamicin and ciprofloxacin among CNA isolates, only isolates that were separately tested against gentamicin and ciprofloxacin were selected. An *Acinetobacter* spp. isolate was defined as ciprofloxacin- or gentamicin-non-susceptible if it was tested intermediate or resistant against ciprofloxacin and gentamicin, respectively.

### Statistical analyses

All statistical analyses were performed using R version 3.5.1 [16] and the “survey” package (version 3.35). Proportions and 95% confidence intervals of carbapenem-non-susceptibility were estimated accounting for clustering at the hospital level and stratified by country level. For all calculations in all strata, country population-based weighting was applied. The population data were obtained from the Eurostat database (https://ec.europa.eu/eurostat/data/database). Weightings were used for each country’s population in the calculation of non-susceptibility proportions in order to ensure that the data contributed proportionally to their population sizes. This was done to minimise biases from significant differences in isolate numbers between the countries. The potential association between different variables and carbapenem-non-susceptibility was analysed using univariable and multivariable logistic regression analyses accounting for clustering and using weights proportional to each country’s population sizes as described above. For the univariable analyses, the following predictors for carbapenem-non-susceptibility were considered: Year of sampling, gender, age group, specimen type, European region, hospital unit type and season. These variables were selected before conducting the analysis and were based on the availability of the data and our prior hypotheses on which variables may be associated with carbapenem-non-susceptibility in *Acinetobacter* spp. We included all variables from the univariable analyses in the model for the multivariable analysis.

## Results

### Dataset characteristics

The baseline characteristics of the analysed *Acinetobacter* spp. isolates are outlined in Table 1. In total, 18,412 isolates of *Acinetobacter* spp. from 18,167 patients were collected in 1191 hospitals across Europe between 2013 and 2017. The majority of isolates originated from elderly patients (median age: 64 years). For the isolates with a reported patient gender (*n* = 12,678), the female/male ratio was 0.72. The clinical samples were predominantly from blood specimens (97%). Isolates were most frequently collected from patients treated on ICUs (41%) and internal medicine units (25%), followed by surgical units (11%) and oncology units (5%). About three-quarters of the isolates (73%) were from the Southern and Eastern regions of Europe, which represented 45% of the mean population of the 30 countries included in the study.

**Table 1:** Baseline characteristics of analysed invasive *Acinetobacter* spp. isolates

	Europe (total)		Northern region		Western region		Southern region		Eastern region	
*Number of isolates*	18,412		610		4367		10,522		2913	
*Year of sampling*										
2013 (n, %)	3110	16.89	202	33.11	637	14.59	2032	19.31	239	8.20
2014 (n, %)	3363	18.27	105	17.21	706	16.17	2074	19.71	478	16.41
2015 (n, %)	4037	21.93	105	17.21	851	19.49	2386	22.68	695	23.86
2016 (n, %)	3789	20.58	72	11.80	1033	23.65	1876	17.83	808	27.74
2017 (n, %)	4113	22.34	126	20.66	1140	26.10	2154	20.47	693	23.79
*Patient gender*										
Female (n, %)	5317	28.88	300	49.18	1845	42.25	2122	20.17	1050	36.05
Male (n, %)	7361	39.98	309	50.66	2295	52.55	3184	30.26	1573	54.00
NA (n, %)	5734	31.14	1	0.16	227	5.20	5216	49.57	290	9.96
Sex ratio (f/m)	0.72		0.97		0.80		0.67		0.67	
*Patient age*										
< 1 years (n, %)	503	2.73	31	5.08	116	2.66	281	2.67	75	2.57
1–19 years (n, %)	659	3.58	71	11.64	239	5.47	230	2.19	119	4.09
20–39 years (n, %)	1136	6.17	66	10.82	365	8.36	399	3.79	306	10.50
40–59 years (n, %)	2956	16.05	104	17.05	874	20.01	1231	11.70	747	25.64
60–79 years (n, %)	5743	31.19	218	35.74	1746	39.98	2499	23.75	1280	43.94
80+ years (n, %)	2243	12.18	120	19.67	853	19.53	911	8.66	359	12.32
NA (n, %)	5172	28.09	0	0.00	174	3.98	4971	47.24	27	0.93
Age (median, IQR)	64.0	49–76	62.0	36–76	66.0	51–78	65.0	50–76	63	48–73
*Specimen (sampling site)*										
Blood (n, %)	17,818	96.77	591	96.89	4304	98.56	10,108	96.07	2815	96.64
CSF (n, %)	594	3.23	19	3.11	63	1.44	414	3.93	98	3.36
*Hospital unit type*										
Intensive care	7605	41.30	36	5.90	702	16.08	5352	50.86	1515	52.01
Internal medicine	4612	25.05	154	25.25	1452	33.25	2509	23.85	497	17.06
Surgical unit	1936	10.51	71	11.64	415	9.50	1204	11.44	246	8.44
Oncology	919	4.99	46	7.54	458	10.49	267	2.54	148	5.08
Other (n, %)	2554	13.87	155	25.41	1042	23.86	908	8.63	449	15.41
NA (n, %)	786	4.27	148	24.26	298	6.82	282	2.68	58	1.99
*Patients with Acinetobacter spp. isolates (n)*	18,167		603		4249		10,415		2905	
*Hospitals (n)*	1191		139		568		366		118	

*Abbreviations*: *95% CI* 95% confidence interval, *CSF* Cerebrospinal fluid, *IQR* Interquartile range, *NA* Not available

### Proportion and temporal trend of CNA in Europe

Between 2013 and 2017, the population-weighted mean proportion of carbapenem-non-susceptible *Acinetobacter* spp. isolates in Europe was 35.6% (95% CI 29.7–42.0%). However, when disaggregated into regions, the profound Southern-Eastern to Northern-Western gradient previously described in EARS-Net reports [13, 17] became apparent. While the Southern and Eastern regions reported CNA proportions of 75.5% (95% CI 71.2–79.4%) and 71.5% (95% CI 66.7–75.9%), respectively, the Northern and Western regions reported CNA proportions as low as 2.8% (95% CI 1.2–6.0%) and 6.3% (95% CI 4.5–8.9%), respectively (Additional File 1: Additional Table 1). Univariable and multivariable regression analyses confirmed that *Acinetobacter* spp. isolates from Southern and Eastern Europe were more likely to be carbapenem-non-susceptible than isolates from Northern and Western European regions (Table 2).

**Table 2:** Analysis of factors associated with carbapenem-non-susceptibility in invasive *Acinetobacter* spp. isolates

	*Univariable analysis*			*Multivariable analysis*		
	OR	(95% CI)	*p*-value	OR	(95% CI)	p-value
*Year of sampling*						
2013–2017	1.02	(0.88–1.18)	0.844	1.03	(0.94–1.13)	0.571
*European region*						
Northern	1	–	–	1	–	–
Western	2.37	(0.98–5.74)	0.056	2.66	(1.04–6.82)	0.041
Southern	109	(47.2–250)	< 0.001	83.76	(33.79–207.66)	< 0.001
Eastern	88.4	(38.3–204)	< 0.001	75.87	(31.23–184.32)	< 0.001
*Patient gender*						
Female	1	–	–	1	–	–
Male	1.54	(1.36–1.73)	< 0.001	1.17	(1.03–1.33)	0.013
*Patient age*						
< 1 years	0.36	(0.16–0.82)	0.014	0.24	(0.10–0.59)	0.002
1–19 years	0.20	(0.12–0.32)	< 0.001	0.28	(0.16–0.49)	< 0.001
20–39 years	1	–	–	1	–	–
40–59 years	1.36	(1.01–1.84)	0.044	1.25	(0.88–1.77)	0.217
60–79 years	1.20	(0.91–1.58)	0.195	1.21	(0.92–1.56)	0.178
80+ years	0.78	(0.58–1.07)	0.123	1.16	(0.85–1.59)	0.352
*Specimen (sampling site)*						
Blood	1	–	–	1	–	–
CSF	1.42	(1.00–2.01)	0.052	0.78	(0.45–1.35)	0.373
*Hospital unit type*						
Internal medicine	1	–	–	1	–	–
Intensive care	6.62	(5.26–8.33)	< 0.001	3.79	(2.94–4.88)	< 0.001
Oncology	0.49	(0.32–0.74)	< 0.001	0.61	(0.43–0.87)	0.006
Surgery	1.47	(1.16–1.88)	0.002	1.17	(0.85–1.60)	0.341
Other	0.64	(0.47–0.85)	0.003	0.81	(0.62–1.06)	0.13
*Season*						
Warm months	0.83	(0.73–0.94)	0.003	0.86	(0.73–1.02)	0.087
Cold months	1	–	–	1	–	–

*Abbreviations*: *95% CI* 95% confidence interval, *CSF* Cerebrospinal fluid, *OR* Odds ratio

**Fig. 1:**
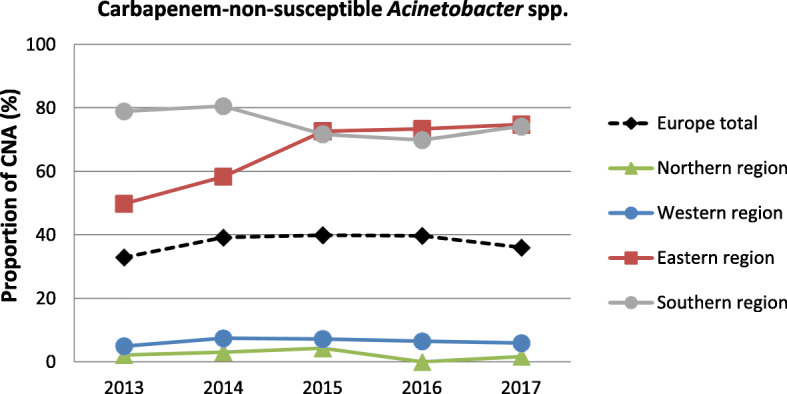
Time trend of carbapenem-non-susceptible *Acinetobacter* spp. Time trend of carbapenem-non-susceptible *Acinetobacter * spp. (CNA) as population-weighted mean proportions (%) among all *Acinetobacter* spp. isolates, with corresponding 95% confidence intervals for the whole of Europe and European regions

For Europe as a whole, the proportion of *Acinetobacter* spp. isolates non-susceptible against carbapenems showed an initial apparent increase between 2013 (32.9% [95% CI 25.8–40.7%]) and 2015 (39.8% [95% CI 32.6–46.0%]), followed by a decline until 2017 (36.0 [95% CI 28.7–44.0%) (Fig. 1 and Additional File 1: Additional Table 1). Subregional analyses showed that CNA proportions remained stable in Northern, Western and Southern European regions. In contrast, CNA proportions increased in Eastern Europe from 49.7% (95% CI 41.0–58.5%) in 2013 to 72.6 (95% CI 67.1–77.4%) in 2015 and remained stable in 2016 and 2017 (Fig. 1 and Additional File 1: Additional Table 1).

### Age and gender

**Fig. 2:**
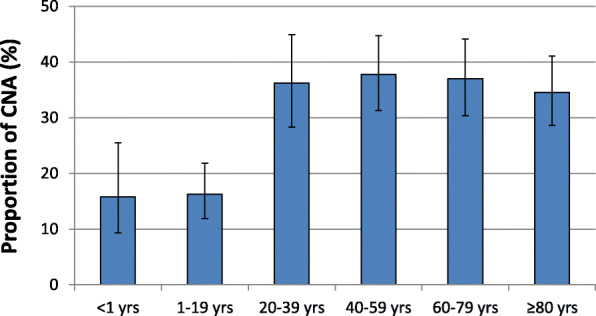
Carbapenem-non-susceptible *Acinetobacter* spp. stratified into age. Carbapenem-non-susceptible *Acinetobacter* spp. (CNA) as a proportion (%) of all *Acinetobacter* spp. isolates, with corresponding 95% confidence intervals for age categories. Yrs: Years

In order to understand the possible influence of the age and gender of patients on CNA infections, these two factors were tested for any association with carbapenem-non-susceptibility. The population-weighted mean proportions for the whole of Europe show that isolates from infants and babies (0–1 years) as well as from children and adolescents (1–20 years) exhibited markedly lower CNA proportions (15.8% [95% CI 9.3–25.5%] and 16.2% [95% CI 11.9–21.8], respectively) compared to older age groups, where > 30% of all *Acinetobacter* spp. isolates were carbapenem-non-susceptible (Fig. 2 and Additional File 1: Additional Table 1). This finding is supported by univariable and multivariable analyses, which confirmed that *Acinetobacter* spp. isolates from patients aged 0–1 years and 1–20 years are less likely to be carbapenem-non-susceptible than isolates from older patients (Table 2). Importantly, similar patterns of relative age differences were observed in Western, Eastern and Southern European regions (Additional File 1: Additional Table 1), irrespective of the overall mean CNA proportion reported in these regions. There was an overall population-weighted mean difference of 3% in the proportion of CNA among isolates between female (32.8% [95% CI 26.9–39.2%]) and male (35.9% [95% CI 29.7–42.6%]) patients (Additional File 1: Additional Table 1). Univariable and multivariable analyses show that male patients have a slightly higher likelihood of CNA than female patients (Table 2).

### Clinical specimen

Analysis by specimen (sampling site) indicates a higher carbapenem-non-susceptibility proportion in CSF isolates (41.0% [95% CI 35.1–47.2%]) than observed in blood isolates (35.6% [95% CI 29.6–42.0%]) (Additional File 1: Additional Table 1). However, only 3% of the isolates were from CSF, and no statistically significant association between specimen type and the likelihood of CNA infection was found in univariable and multivariable regression analyses (Table 2).

### Hospital unit type

**Fig. 3:**
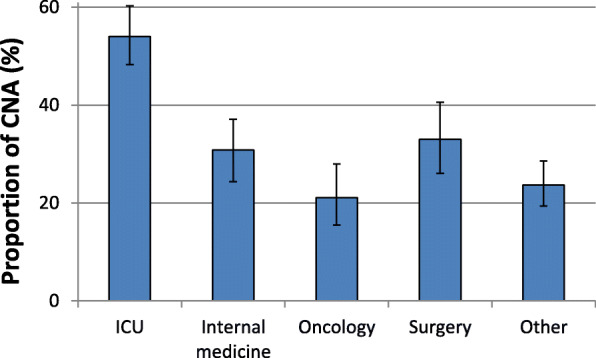
Carbapenem-non-susceptible *Acinetobacter* spp. stratified by hospital unit types. Carbapenem-non-susceptible *Acinetobacter* spp. (CNA) as population-weighted mean proportions (%) among all *Acinetobacter* spp. isolates, with corresponding 95% confidence intervals for hospital unit types. ICU: Intensive care unit

Analyses of various hospital unit types where the samples were collected reveal substantial differences in CNA proportions (Fig. 3 and Additional File 1: Additional Table 1). For the whole population-weighted European sample set, ICUs recorded a significantly higher proportion of CNA (54.0% [95% CI 47.6–60.3%]) compared to other hospital units. Internal medicine units and surgical wards recorded CNA proportions of 30.8% (95% CI 25.1–37.1%) and 33.0 (95% CI 26.1–40.6%), respectively. Univariable and multivariable regression analyses confirmed that the likelihood of CNA on ICUs is markedly more likely than on other hospital units (Table 2). The likelihood of CNA acquisition on oncological units is lower than on internal medicine units (OR: 0.61 [95% CI 0.43–0.87, *p* = 0.006]. Substantially higher CNA proportions of invasive *Acinetobacter* spp. isolates collected from ICUs in relation to other hospital units were observed across all European regions (Additional File 1: Additional Table 1).

### Seasonality

For Europe as a whole, CNA proportions did not differ between samples that were collected during the “Cold months” (36.4% [95% CI 30.4–43.0%] and “Warm months” (34.6% [95% CI 28.7–41.0%]) (Additional File 1: Additional Table 1). No statistically significant association between seasonality and CNA proportions was therefore found in multivariable analyses (OR: 0.86 [95% CI 0.73–1.02], *p* = 0.087) (Table 2). Moreover, there were no major differences in CNA proportions between cold and warm months on European regional level (Northern: 2.8% [95% CI 0.9–7.9%] vs. 2.8% [95% CI 0.9–8.6%]; Western: 6.4% [95% CI 4.5–9.0%] vs. 6.4% [95% CI 4.4–9.1%]; Southern: 77.3% [95% CI 72.6–81.4%] vs. 73.3% [95% CI 60.5–77.7%]; Eastern: 73.7% [95% CI 68.1–78.6%] vs. 68.9% [95% CI 64.2–73.2%].

### Co-resistance

Since fluoroquinolones (e.g. ciprofloxacin) and aminoglycoside antibiotics (e.g. gentamicin) represent potential therapy options to treat CNA infections, ciprofloxacin and gentamicin resistance proportions among carbapenem-susceptible and carbapenem-non-susceptible *Acinetobacter* spp. isolates were studied. The majority of *Acinetobacter* spp. isolates that are non-susceptible to carbapenems also exhibited non-susceptibility to ciprofloxacin (65.1% [95% CI 56.7–72.6%]) and gentamicin (81.0% [95% CI 77.1–84.4%]). In contrast, among carbapenem-susceptible *Acinetobacter* strains, ciprofloxacin- and gentamicin non-susceptibility proportions were 3.4% [95% CI 2.6–4.4%] and 14.7% [95% CI 10.9–19.5%]), respectively. Importantly, co-resistance to ciprofloxacin among CNA is particularly high in Southern and Eastern European regions (95.0% [95% CI 93.5–96.2%]) and 87.0% (95% CI 82.1–90.7%], respectively. Similarly, both regions also exhibit a high burden of gentamicin co-non-susceptibility among carbapenem-non-susceptible *Acinetobacter* isolates (Southern: 96.1% [95% CI 94.6–97.2%], Eastern: 89.4% [95% CI 86.6–91.6%]).

## Discussion

In this study, we assessed epidemiological trends of invasive carbapenem-non-susceptible *Acinetobacter* spp. isolates from hospital patients in the European Union and the European Economic Area (EAA) using data from EARS-Net. Similar to the conclusions reached previously by the ECDC [1], our study demonstrated the persisting challenge of CNA in Europe as the country-population weighted mean proportion of non-susceptible isolates exceeds 30% for the period between 2013 and 2017. The CNA proportion observed in our study is similar to the CNA proportion (35%) reported in a recently published nationwide survey done in the United States among hospitalized patients [18]. A recent global study showed that carbapenem-non-susceptibility in Acinetobacter baumannii complex isolates from both invasive and non-invasive infections was considerably higher in the Asia-Pacific region (~ 79%), Latin America (~ 85%) and North America (~ 45%) compared to the mean CNA proportion in Europe described in our study [19]. Importantly, in Europe the Southern-Eastern to Northern-Western gradient persists. In the Southern and Eastern regions, more than 70% of all *Acinetobacter* spp. isolates from invasive infections were carbapenem-non-susceptible, while in the North and the West, CNA proportions of less than 10% were observed. This gradient seen in the non-susceptibility of invasive *Acinetobacter* spp. to carbapenems was also observed for other pathogens in Europe, including *Pseudomonas aeruginosa* and *Klebsiella pneumoniae* [13], suggesting a systematic higher burden of invasive infections with drug-resistant Gram-negative pathogens in Southern and Eastern European regions. High CNA proportions in the Southern and Eastern regions is of great clinical concern, particularly in light of growing resistance against other antimicrobial drugs used as alternative therapeutic options to treat CNAs infections, such as fluoroquinolones and aminoglycoside antibiotics. Our analyses show that on average, > 70% of CNA are also non-susceptible to ciprofloxacin or gentamicin in Southern and Eastern Europe.

Differences in antimicrobial use, infection control and health care utilisation practices between the countries are possible reasons for the variation in the reported proportion of drug-resistant *Acinetobacter* spp. isolates [20]. Previous studies have shown limited organizational support and practice of infection control measures like standard precautions among health workers that work in the medical ward as well as in critical areas like the ICU in Eastern (e.g. Poland) and Southern European (e.g. Greece) countries [21–23]. The 2017 ESAC-Net report showed an increasing trend of carbapenem consumption between 2013 and 2017 in many of the Southern and Eastern region countries, including Latvia, Lithuania, Romania, Poland and Hungary [24]. This might partly explain the surge in carbapenem-non-susceptibility seen in these regions. In fact, an association between hospital carbapenem consumption and carbapenem-resistance rates of *Acinetobacter* have been described by multiples studies [25–29]. Beside antibiotic usage, other possible explanations for the observed gradient in CNA proportion have been suggested, such as differences in climate and socio-economic conditions across these European regions [30, 31].

In addition to regional differences in CNA proportions in Europe, this study identified factors that are associated with a higher likelihood of acquisition of carbapenem-non-susceptible *Acinetobacter* spp. Adult patients (> 20 years) show markedly higher CNA proportions than both babies and infants (< 1 years) and children and adolescents between 1 and 20 years. Importantly, the differences in non-susceptibility proportion between adults and babies/adolescents is also observed in regional analyses in Western, Southern and Eastern European regions, suggesting that the higher proportion of CNA in adult patients is a common feature in invasive *Acinetobacter* spp. infections, independent of inter-regional differences in CNA proportions. This finding is consistent with other studies that found children and adolescents are less susceptible to several infections with drug-resistant bacteria, including carbapenem-non-susceptible *Klebsiella pneumoniae* [32], *Pseudomonas aeruginosa* [33], as well as methicillin-resistant *Staphylococcus aureus* [34] and vancomycin-resistant *Enterococcus faecium* [35, 36]. Several factors may explain these age differences. It has been shown that elderly patients are more likely to be colonised with drug-resistant pathogens due to more frequent exposure to antibiotics throughout their lives. Moreover, elderly patients have more comorbidities than younger patients and are more likely to reside in nursing homes or other healthcare facilities, both factors that have been shown to be associated with increased AMR [37]. This study also shows that male patients are associated with an increased likelihood of CNA compared to female patients. Male gender is also associated with higher proportions of other carbapenem-non-susceptible pathogens, including of *Klebsiella pneumoniae* and other Gram-negative rods [32, 38].

Unsurprisingly, most of the isolates in this study are from the ICUs, which is a known hotbed for nosocomial transmission of *Acinetobacter* spp. [11, 39]. This study shows that the proportion of CNA is highest among isolates collected in the ICU where many patients tend to have severe comorbidities and are exposed to antibiotics and devices that enable the transmission of pathogens [40–42]. Studies have also reported that admission to the ICU is a risk factor for the acquisition and isolation of CNA infections in other regions of the world, such as Northern Africa [43, 44].

### Strengths and limitations

To our knowledge, this study is the largest and most comprehensive analysis of CNA infections in Europe, with more than 18,000 clinical isolates from 30 countries in the EU/EAA. EARS-Net data are based on routine clinical antimicrobial susceptibility data from national surveillance programmes. The accuracy and validity of the AMR data has been assured through annual external quality assessments on all laboratories from all countries, which showed they all performed generally well [45]. However, it is important to consider the limitations of EARS-Net data. Firstly, participating national laboratories and hospitals might not be representative of an individual country, although according to the EARS-Net report 2017 [13], many countries across all regions reached a high isolate sample representativeness in their national surveillance systems. Secondly, population coverage varied among reporting countries. To address this issue, all statistical analyses used weightings based on the population sizes of the individual countries similar to that done in the EARS-Net reports. Thirdly, *Acinetobacter spp.* are not identified to the species level in the EARS-Net database. This is important in terms of carbapenem resistance, as *A. baumannii* isolates are more frequently carbapenem-resistant that other *Acinetobacter* species. Therefore, the differential proportion of *A. baumannii* among all *Acinetobacter* species across countries and regions might impact the CNA proportions recorded in EARS-Net. Last, different sampling routines in different healthcare settings such as due to limited resources can result in biased estimates of CNA proportions across European regions. However, due its clinical significance, inclusion of only invasive clinical specimen limits the bias that may result from some of the inconsistencies in sampling, although frequency of blood sampling varies between hospitals and countries.

## Conclusion

Carbapenem-non-susceptible *Acinetobacter* spp. pose a significant threat to public health in Europe, particularly in the Southern and Eastern regions where more than 70% of all invasive *Acinetobacter* isolates were non-susceptible to carbapenems. The proportion of CNA is particularly high on ICUs and among older patients. These findings raise concern for inter-regional spread to areas with traditionally low CNA proportions from contiguous European countries with high CNA proportions. In order to prevent the spread of CNA infections, continued surveillance and implementation of effective infection prevention and control measures are needed, particularly in high-risk settings and through targeting high risk groups.

## Supplementary information

**Additional file 1: Additional Table 1.** Proportions (%) and 95% confidence intervals of carbapenem-non-susceptible Acinetobacter spp. isolates.

## Data Availability

Raw data were obtained from the TESSy database (EARS-Net) with the approval of the ECDC. All raw data can be provided on reasonable request with the approval of the ECDC.

## Abbreviations

AMR: Antimicrobial Resistance
AST: Antimicrobial susceptibility testing
CI: Confidence intervals
CLSI: Clinical & Laboratory Standards Institute
CNA: Carbapenem-non-susceptible *Acinetobacter* spp.
CSF: Cerebrospinal fluid
EARS-Net: European Antimicrobial Resistance Surveillance Network
ECDC: European Centre for Disease Prevention and Control
EUCAST: European Committee on Antimicrobial Susceptibility Testing
ICU: Intensive care unit
IQR: Interquartile range
OR: Odds ratio
WHO: World Health Organization
