# Supplementary material for: The epidemiology of carbapenem-non-susceptible Acinetobacter species in Europe: analysis of EARS-Net data from 2013 to 2017
Source: Antimicrob Resist Infect Control. 2020 Jun 19;9:89. doi: 10.1186/s13756-020-00750-5 (PMC7304165; doi:10.1186/s13756-020-00750-5)
Supplement: Supplementary file 1 — Additional file 1: Additional Table 1. Proportions (%) and 95% confidence intervals of carbapenem-non-susceptible Acinetobacter spp. isolates. [file 13756_2020_750_MOESM1_ESM.docx]

**Additional Files**

| **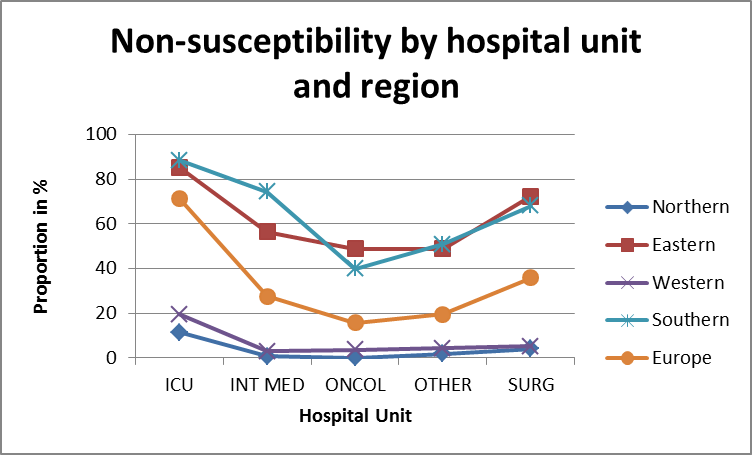Additional Table 1.** Proportions (%) and 95% confidence intervals of carbapenem-non-susceptible *Acinetobacter* spp. isolates | | | | | | | | | | | | | | | |
| --- | --- | --- | --- | --- | --- | --- | --- | --- | --- | --- | --- | --- | --- | --- | --- |
|  |  | Europe  (total) | |  | Northern  region | |  | Western  region | |  | Southern  region | |  | Eastern  region | |
|  |  | R+I (%) | (95% CI) |  | R+I (%) | (95% CI) |  | R+I (%) | (95% CI) |  | R+I (%) | (95% CI) |  | R+I (%) | (95% CI) |
| *Year of sampling* | | | |  |  |  |  |  |  |  |  |  |  |  |  |
|  | 2013 | 32.9 | (25.8-40.7) |  | 3.1 | (1.1-8.1) |  | 7.4 | (4.4-12.2) |  | 78.8 | (71.3-84.8) |  | 49.7 | (41.0-58.5) |
|  | 2014 | 39.1 | (32.6-46.0) |  | 2.2 | (0.4-10.2) |  | 5.0 | (2.0-11.9) |  | 80.5 | (72.4-86.7) |  | 58.3 | (45.6-69.9) |
|  | 2015 | 39.8 | (32.8-47.4) |  | 4.3 | (1.7-10.2) |  | 7.2 | (4.9-10.5) |  | 71.7 | (64.5-77.9) |  | 72.6 | (67.1-77.4) |
|  | 2016 | 39.7 | (32.7-47.0) |  | 0.0 | (-) |  | 6.5 | (4.3-9.8) |  | 69.9 | (62.1-76.6) |  | 73.3 | (68.4-77.6) |
|  | 2017 | 36.0 | (28.7-44.0) |  | 1.7 | (0.2-12.0) |  | 5.9 | (4.4-8.0) |  | 74.1 | (66.8-80.3) |  | 74.7 | (69.6-79.2) |
|  | 2013-2017 | 35.6 | (29.7-42.0) |  | 2.8 | (0.1-6.0) |  | 6.3 | (4.5-8.9) |  | 75.5 | (71.2-79.4) |  | 71.5 | (66.7-75.9) |
| *Patient gender* | | | |  |  |  |  |  |  |  |  |  |  |  |  |
|  | Female | 32.8 | (26.9-39.2) |  | 2.7 | (0.9-7.5) |  | 5.4 | (3.6-8.0) |  | 69.4 | (63.9-74.4) |  | 68.3 | (62.4-73.7) |
|  | Male | 35.9 | (29.7-42.6) |  | 2.8 | (0.8-9.0) |  | 7.4 | (2.0-7.6) |  | 75.0 | (70.4-79.2) |  | 73.0 | (68.6-77.1) |
| *Patient age* | | | |  |  |  |  |  |  |  |  |  |  |  |  |
|  | <1 years | 15.8 | (9.3-25.5) |  | 12.6 | (2.1-48.7) |  | 3.8 | (1.2-11.4) |  | 30.0 | (13.8-53.4) |  | 22.2 | (10.9-39.9) |
|  | 1-19 years | 16.2 | (11.9-21.8) |  | 0.0 | (-) |  | 3.6 | (1.5-8.0) |  | 29.5 | (16.8-46.4) |  | 41.8 | (32.4-51.8) |
|  | 20-39 years | 36.2 | (28.3-44.9) |  | 4.8 | (0.9-21.0) |  | 9.5 | (6.2-14.3) |  | 69.2 | (58.2-78.4) |  | 77.1 | (71.7-81.7) |
|  | 40-59 years | 37.8 | (31.3-44.7) |  | 1.0 | (0.1-7.2) |  | 8.4 | (5.2-13.2) |  | 79.7 | (74.4-84.2) |  | 75.1 | (67.1-81.7) |
|  | 60-79 years | 37.0 | (30.4-44.2) |  | 3.5 | (1.0-12.0) |  | 6.2 | (4.1-9.4) |  | 78.9 | (73.9-83.1) |  | 73.6 | (69.6-77.2) |
|  | 80+ years | 34.5 | (28.6-41.0) |  | 0.0 | (-) |  | 4.3 | (3.0-6.3) |  | 76.4 | (69.6-82.1) |  | 71.1 | (65.6-76.0) |
| *Specimen (sampling site)* | | | |  |  |  |  |  |  |  |  |  |  |  |  |
|  | Blood | 35.6 | (29.6-42.0) |  | 2.7 | (1.2-6.0) |  | 6.3 | (4.4-8.9) |  | 75.6 | (71.2-79.5) |  | 71.4 | (66.4-75.9) |
|  | CSF | 41.0 | (35.1-47.2) |  | 0.9 | (0.1-8.4) |  | 7.4 | (3.0-17.5) |  | 73.3 | (62.3-82.0) |  | 77.0 | (68.3-83.9) |
| *Hospital unit type* | | | |  |  |  |  |  |  |  |  |  |  |  |  |
|  | Internal medicine | 30.8 | (25.1-37.1) |  | 0.5 | (0.1-2.0) |  | 2.4 | (1.4-4.3) |  | 72.2 | (65.9-77.7) |  | 58.4 | (50.2-66.2) |
|  | Intensive care | 54.0 | (47.6-60.3) |  | 22.2 | (17.3-8.1) |  | 20.7 | (15.5-26.9) |  | 87.9 | (84.0-90.9) |  | 83.2 | (78.7-86.9) |
|  | Oncology | 21.1 | (15.5-28.0) |  | 0.0 | (-) |  | 3.7 | (1.9-7.0) |  | 38.3 | (22.9-56.5) |  | 54.9 | (45.8-63.7) |
|  | Surgery | 33.0 | (26.1-40.6) |  | 1.4 | (0.3-7.3) |  | 4.7 | (2.4-9.1) |  | 68.7 | (60.5-75.8) |  | 73.5 | (65.2-80.4) |
|  | Other | 23.7 | (19.4-28.6) |  | 1.1 | (0.1-8.4) |  | 4.2 | (2.5-6.9) |  | 52.4 | (43.4-61.3) |  | 44.6 | (36.8-52.8) |
| *Season* | | | |  |  |  |  |  |  |  |  |  |  |  |  |
|  | Cold months | 36.5 | (30.4-43.0) |  | 2.8 | (0.9-7.9) |  | 6.4 | (4.5-9.0) |  | 77.3 | (72.6-81.4) |  | 73.7 | (68.1-78.6) |
|  | Warm months | 34.6 | (28.7-41.0) |  | 2.8 | (0.9-8.6) |  | 6.4 | (4.4-9.1) |  | 73.3 | (68.5-77.7) |  | 68.9 | (64.2-73.2) |
| *Abbreviations: 95% CI: 95% confidence interval; CSF: Cerebrospinal fluid; R+I: Tested as resistant or intermediate in antimicrobial susceptibility testing* | | | | | | | | | | | | | | | |
